# Antimicrobial Use Survey and Detection of ESBL-*Escherichia coli* in Commercial and Medium-/Small-Scale Poultry Farms in Selected Districts of Zambia

**DOI:** 10.3390/antibiotics13050467

**Published:** 2024-05-20

**Authors:** Taona Sinyawa, Misheck Shawa, Geoffrey M. Muuka, Fusya Goma, Paul Fandamu, Joseph Yamweka Chizimu, Cynthia Sipho Khumalo, Malala Mulavu, Masuzyo Ngoma, Herman Moses Chambaro, Harvey Kakoma Kamboyi, Masahiro Kajihara, Hirofumi Sawa, Yasuhiko Suzuki, Hideaki Higashi, Geoffrey Mainda, Musso Munyeme, John Bwalya Muma, Christian Owusu Nyantakyi, Beverly Egyir, Bernard Mudenda Hang’ombe

**Affiliations:** 1 Central Veterinary Research Institute, Ministry of Fisheries and Livestock, Chilanga, Lusaka 10101, Zambia; taonasinyawa@gmail.com (T.S.); iamisinme@gmail.com (M.N.); hermcham@gmail.com (H.M.C.); 2 Hokudai Centre for Zoonosis Control in Zambia, University of Zambia, Lusaka 10101, Zambia; misheckshawa@czc.hokudai.ac.jp (M.S.); kajihara@czc.hokudai.ac.jp (M.K.); h-sawa@ivred.hokudai.ac.jp (H.S.); 3 Department of Veterinary Services, Ministry of Fisheries and Livestock, Lusaka 15100, Zambia; muuka.geoffrey@mfl.gov.zm (G.M.M.); fandamu.paul@mfl.gov.zm (P.F.); 4 Zambia National Public Health Institute, Stand 1186, Coner of Chaholi and Addis Ababa Roads, Rhodes Park, Lusaka 10101, Zambia; chizimuyjoseph@yahoo.com; 5 Department of Biomedical Sciences, School of Veterinary Medicine, University of Zambia, Lusaka 10101, Zambia; khumalocynthiasipho@gmail.com; 6 Department of Biomedical Sciences, School of Health Sciences, University of Zambia, Lusaka 10101, Zambia; m.mulavu@gmail.com; 7 Division of Infection and Immunity, International Institute for Zoonosis Control, Hokkaido University, N20 W10, Kita-ku, Sapporo 001-0020, Japan; kamboyihk@czc.hokudai.ac.jp (H.K.K.); hidea-hi@czc.hokudai.ac.jp (H.H.); 8 Division of International Research Promotion, International Institute for Zoonosis Control, Hokkaido University, N20 W10, Kita-ku, Sapporo 001-0020, Japan; 9 Institute for Vaccine Research and Development (HU-IVReD), Hokkaido University, N21 W11, Kita-ku, Sapporo 001-0020, Japan; 10 Division of Bioresources, International Institute for Zoonosis Control, Hokkaido University, N20 W10, Kita-ku, Sapporo 001-0020, Japan; 11 Food and Agriculture Organization of the United Nations (FAO), Chaholi Road, Rhodes Park, Lusaka 10101, Zambia; geoffrey.mainda@fao.org; 12 Department of Disease Control, School of Veterinary Medicine, University of Zambia, Lusaka 10101, Zambia; mmunyeme@unza.zm (M.M.); jmuma@unza.zm (J.B.M.); 13 Bacteriology Department, Noguchi Memorial Institute for Medical Research, College of Health Sciences, University of Ghana, Accra 00233, Ghana; cowusu-nyantakyi@noguchi.ug.edu.gh (C.O.N.); begyir@noguchi.ug.edu.gh (B.E.); 14 Microbiology Unit, Department of Para-Clinical Studies, Africa Centre of Excellence for Infectious Diseases of Humans and Animals (ACEIDHA), School of Veterinary Medicine, University of Zambia, Lusaka 10101, Zambia

**Keywords:** AMR, AMU, commercial, *Escherichia coli*, ESBL, medium-/small-scale, WGS, Zambia

## Abstract

Antimicrobial resistance (AMR) among *Escherichia coli* from food animals is a rising problem, and heavy antimicrobial use in poultry is a contributing factor. In Zambia, studies linking poultry-associated AMR and antibiotic use (AMU) are rare. This study aimed to investigate commercial and medium-/small-scale poultry farmers’ usage of antimicrobials based on a questionnaire survey in ten districts of Zambia. In addition, the study characterized extended-spectrum *β*-lactamase (ESBL)-producing *E. coli* isolates obtained from poultry in the same districts. Data regarding knowledge and usage of antimicrobials were collected from commercial and medium-/small-scale poultry farmers using a pre-tested structured questionnaire. At the same time, cloacal samples were collected and analyzed. One hundred and fifty *E. coli* isolates were tested for antimicrobial susceptibility using eight antibiotic classes. The isolates were further screened for ESBL production by streaking them on cefotaxime (CTX)-supplemented MacConkey agar, then subjecting them to sequencing on a NextSeq. The questionnaire survey showed that more medium-/small-scale than commercial poultry farmers used antimicrobials (OR = 7.70, 95% CI = 2.88–20.61) but less prescriptions (OR = 0.02, 95% CI = 0.00–0.08). Susceptibility testing revealed that resistance was highest to ampicillin (128/148, 86.5%) and tetracycline (101/136, 74.3%) and that the prevalence of multidrug resistance (MDR) (28/30, 93.3%) was high. Whole-genome sequencing (WGS) of eight (8/30, 26.7%) isolates with CTX Minimum Inhibitory Concentration (MIC) ≥ 4 µg/mL revealed the presence of ESBL-encoding genes *bla*_CTX-M-14_, *bla*_CTX-M-55_, and *bla*_TEM_. WGS also detected other AMR genes for quinolones, aminoglycosides, phenicols, tetracycline, macrolides, and folate-pathway antagonists. Altogether, the questionnaire survey results showed a higher proportion of AMU and lower prescription usage among medium-/small-scale farmers. In addition, our results emphasize the circulation of ESBL-producing *E. coli* strains with associated MDR. It is critical to educate farmers about AMR risks and to encourage responsible usage of antimicrobials. Furthermore, there is a need to strengthen regulations limiting access to antimicrobials. Finally, there is a need to establish a one health system to guide public health response.

## 1. Introduction

Antimicrobials play an essential role in human and food-animal health and represent one of the main therapeutic tools for human and veterinary medicine [1]. As a result, antimicrobial resistance (AMR) has emerged as a global public health concern. The Centers for Disease Control and Prevention (CDC), in a 2019 report, summarized that humanity would face increasingly resistant infections, potentially extending to all treatments available, leading to what is coined the “post-antibiotic era” [2].

While AMR can take many forms, extended-spectrum *β*-lactamases (ESBLs) have great clinical significance in medical bacteriology as they threaten both therapeutics from antimicrobials and infection control in humans and animals [3]. ESBLs are a rapidly evolving group of *β*-lactamases that confer resistance to most *β*-lactam antibiotics, including penicillin, third-generation cephalosporins, and the monobactam aztreonam by hydrolyzing their *β*-lactam ring yet can be inhibited by clavulanic acid [4]. Typically, they derive from the narrow spectrum *β*-lactamases, TEM-1, TEM-2, and SHV-1 that usually give rise to ESBLs through point mutations. However, a relatively recent group, the CTX-M type, has become more dominant. The most common *β*-lactamases in gram-negative bacteria are TEM, SHV, OXA, CMY, and CTX-M, and these are encoded by the *bla* genes *bla*_TEM_ *bla*_SHV_, *bla*_OXA_, *bla*_CMY_, and *bla*_CTX-M_, respectively [5]. ESBLs are often plasmid-encoded, and these plasmids frequently carry genes encoding resistance to other drug classes. Therefore, antibiotic options in the treatment of ESBL-producing organisms are extremely limited [6].

The zoonotic potential of most ESBL-producing organisms is a significant public health concern. While the selection of AMR is often associated with hospital antibiotic use, many animal reservoirs now exist. There are several reports of drug-resistant *Enterobacteriaceae* in various livestock, including poultry, sheep, cattle, and pigs [7,8,9,10]. Furthermore, despite the lack of prior antibiotic exposure in wildlife, AMR has been reported in monkey [11], green sea turtles [12], and black rhinoceros [13], probably due to exposure to antibiotic-resistant organisms at the human–animal–environment interface. AMR is a threat to humans and livestock because of the inappropriate use of antibiotics and the use of antibiotics as growth promoters in food animals.

In food animal production, poultry is one of the most widespread types of meat produced and consumed worldwide. Poultry product consumption trends generally show greater demand and wider acceptance across socio-economic, cultural, religious, and other barriers than any other meat of animal origin [14]. Its approval is also anchored on its relative affordability, with prices ranging from USD2.0 to USD3.5 per kg. Furthermore, poultry production is attractive as it requires less financial capital and takes less time to reach the point of sale. Zambia’s poultry sector has seen consistent growth due to the rising human population and concomitant increased demand for chicken meat and eggs, with a bird stock close to 15.5 million. This industry contributes 5% of the nation’s Gross Domestic Product [15].

The increasing demand for poultry products has pressured farmers to enhance production. In some cases, this has led to irrational use of antimicrobials, such as during the treatment of infectious diseases, prophylaxis, meta-phylaxis, and growth promotion [16]. Furthermore, less than 20% of households engaged in poultry production have access to veterinary services, a situation likely to promote the abuse of antimicrobials through self-prescriptions and treatment [14]. Unlike antimicrobials for treatment, the use for growth promotion is usually at subtherapeutic amounts, which may drive the development of AMR [17]. Abuse of antimicrobials results in continuous exposure of an animal’s (the avian) intestinal flora to antimicrobials, creating a selection pressure that leads to AMR [18,19].

In Zambia, poultry is one of the most studied sectors regarding ESBLs. Since the first report by Chishimba et al., 2016 [20], many studies have been published with ESBL prevalence ranging from 3.4% to 20.1%. The commonly isolated ESBL-producing organism is *E. coli* [21,22,23], with sequence types (STs) 55 and 69 reported so far. Furthermore, Zambian strains of poultry origin show that the *bla*_CTX-M_, gene is the commonest ESBL gene, but *bla*_TEM_ and *bla*_SHV_ have also been reported. Importantly, a recent report in Zambia found a link between multidrug resistance in (MDR) *Escherichia coli* (*E. coli*) from humans and poultry [24], suggesting that poultry could be a reservoir. Still, despite these reports, the extent of the problem in poultry remains unclear. Additionally, the relationship between AMR and antibiotic usage in poultry has not been explored. This study reports ESBL patterns in commercial and medium-/small-scale poultry farms of selected districts in Zambia and relates this to antimicrobial use.

## 2. Results

### 2.1. Antibiotic Use (AMU) Differed between Commercial and Medium-/Small-Scale Poultry Farmers

A total of 119 poultry farmers participated in this study, with 84 being medium-/small-scale farmers and 35 being commercial farmers. Only 38.9% (42/108) of the respondents indicated having acquired a prescription before accessing antibiotics. Further, while most farmers acknowledged using antibiotics for treatment, 19.3% (23/119) and 12.6% (15/119) used them for prevention and growth promotion, respectively. Meanwhile, 87.3% (103/118) of the farmers expressed knowledge of the antibiotic withdrawal period. Nonetheless, 5.8% (6/103) of the farmers who claimed to be knowledgeable farmers sold their meat and egg products under treatment (Table 1).

We compared the questionnaire-based variables among different production categories and farming scales to determine the factors associated with the practices mentioned above. Compared to commercial farmers, more medium-/small-scale farmers used antibiotics on the sampled birds in general (Table 1) (OR = 7.70, 95% CI = 2.88–20.61) and for prophylaxis (OR = 12.06, 95% CI = 1.56–93.45). However, medium-/small-scale farmers were less likely to obtain prescriptions (OR = 0.02, 95% CI = 0.00–0.08). Furthermore, while there was no antibiotic use for production among commercial farmers, 17.9% (15/84) of medium-/small-scale farmers used antimicrobial growth promoters. Despite the observed differences in practices towards antibiotic usage, there was no difference in knowledge of the antibiotic withdrawal period between the two groups (OR = 0.55, 95% CI = 0.15–2.10). Likewise, there was no difference in the proportion of farmers selling products from birds under antibiotic treatment (OR = 1.68, 95% CI = 0.44–6.42).

According to respondent information, antimicrobial use among broiler and layer farmers revealed that more layer farmers compared to broiler farmers used antibiotics on the sampled birds (OR = 4.23, 95% CI = 1.54–11.63) for growth promotion (OR = 6.29, 95% CI = 2.00–19.77). On the contrary, there was no difference between farmers who reared layers or broilers in obtaining prescriptions to access antibiotics (OR = 1.23, 95% CI = 0.47–3.23). Additionally, there was no difference in the usage of antibiotics for disease prevention between broiler and layer farmers (OR = 2.67, 95% CI = 0.97–7.34) or those who were acquainted with the period of withdrawal (OR = 0.96, 95% CI = 0.25–3.74) (Supplementary Materials Table S1). However, compared to broiler farmers, more layer farmers sold their products during treatment (OR = 5.38, 95% CI = 1.66–17.42).

### 2.2. Antimicrobial Resistance of E. coli Isolates

**Figure 1:**
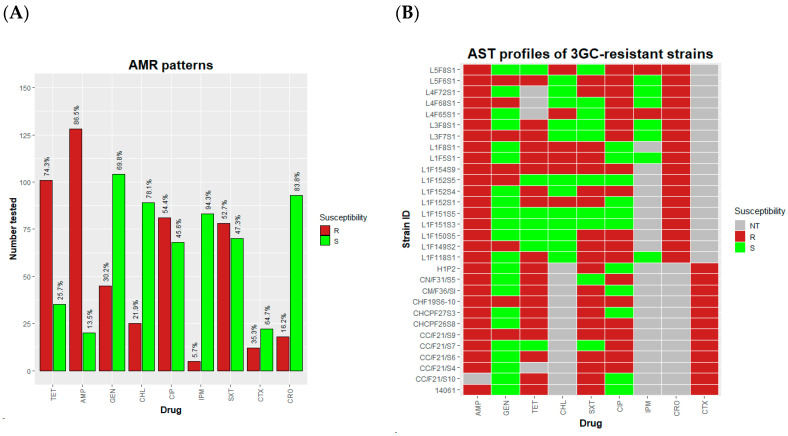
AMR patterns of *E. coli* isolates. (**A**). Percentage resistance by antibiotic. (**B**). AST profiles by isolate. TET-tetracycline, AMP-ampicillin, GEN-gentamicin, CHL-chloramphenicol, IMP-imipenem, SXT-sulfamethoxazole/trimethoprim, CIP-ciprofloxacin, CTX-cefotaxime, CRO-ceftriaxone. R-Resistant, S-Susceptible, NT-Not tested.

In Zambia, all antibiotics are imported into the country. Tetracyclines and penicillins are among the most imported antibiotics for animal administration [25]. To determine if the AMR profiles of poultry-associated *E. coli* were related to the national veterinary antibiotic import data, we subjected the strains to antimicrobial susceptibility testing (AST). Our results showed the highest resistance to AMP (128/148, 86.5%) and TET (101/136, 74.3%) (Figure 1A). On the other hand, imipenem revealed the lowest non-susceptibility (5/88, 5.7%). Third-generation cephalosporin (3GC) resistance was detected in 20% (30/150) of the isolates, most of which (28/30, 93.3%) exhibited resistance to three or more antibiotic classes.

### 2.3. Phenotypic ESBL Prevalence Varied by Production Scale

A total of 150 *E. coli* isolates were screened for cefotaxime resistance by broth microdilution. Thirty isolates met the Minimum Inhibitory Concentration (MIC) breakpoint for cefotaxime resistance (≥2 µg/mL). The results showed a higher proportion of phenotypic ESBL positives among medium-/small-scale farmers, 73% (22/30) compared to 27% (8/30) commercial farms. Chilanga district had the highest number of isolates with phenotypic ESBL positives at 2/3 (66.7%), followed by Chongwe districts at 6/12 (50%) and Petauke at 3/10 (30.0%). On the other hand, no ESBLs were detected in Rufunsa and Chibombo (Supplementary Materials Table S2).

### 2.4. Whole-Genome Sequence Characteristics of ESBL

Eight out of nine strains were successfully assembled into nearly complete genomes with the exception of one strain that had poor quality reads. These eight isolates carried a total of 27 different types of AMR genes that encode resistance to eight classes of antimicrobials (Table 2). The eight sequenced isolates belonged to six different sequence types (ST); ST770 (3/8, 37.5%) was detected three times (2/8, 25%) in Chisamba and (1/8, 12.5%) in Petauke, while 5 STs were assigned as singletons (Table 3). In addition, a diversity of plasmid replicons was observed across the strains, with incompatibility group F dominating (Table 3).

## 3. Discussion

Antimicrobial resistance is fueled by the misuse and abuse of antimicrobial drugs [26]. In this study, we report questionnaire survey information on AMU, and phenotypic and genotypic characterization of isolated *E. coli* strains. The survey results showed that broiler and layer poultry farmers in Zambia use antimicrobials, with nearly 50% of the sampled birds being treated with antibiotics. The results also show that more medium-/small-scale than commercial poultry farmers used antimicrobials for prophylaxis. Additionally, the study demonstrated that farmers utilized antimicrobials to promote growth more frequently in layers than in broiler poultry. Finally, the laboratory results showed 20% of the isolates were resistant to 3GCs, associated with *bla*_CMY_, *bla*_CTX-M_, and *bla*_TEM_ genes.

While some countries report higher than 90% AMU [27,28], our questionnaire survey highlighted an overall 48.3% (57/118) AMU among poultry farmers, similar to another Zambian survey report by Caudell et al. (2020) [29]. However, Caudell et al. (2020) also reported 80% (158/198) lack of obtaining a prescription when purchasing antimicrobials compared to nearly 60% reported (66/108) (OR = 2.51, 95% CI = 1.49–4.23) in our study. This discrepancy could be attributed to our study covering three more provinces and five more districts [29] and possible temporal variations. Although this study shows lower AMU than other nations, the need to use antimicrobials among these farmers could be exacerbated by poor implementation of biosecurity measures, leading to increased AMU for disease prevention [30]. Therefore, it is necessary to improve farm-level infection management practices, including vaccines [31], phytogenic feed additives, and bioactive phenolic extracts [32], among other available solutions.

Questionnaire data analysis reported no difference in AMU for growth promotion between commercial and medium-/small-scale farmers (OR = 7.61, CI = 0.97–59.98), suggesting that any difference in the overall usage could be related to infection management. Considering that commercial farmers have more established systems in terms of biosecurity [33] and thus experience fewer infections, AMU under these conditions is expected to be lower. Consistently, we observed a significant difference in AMU between commercial and medium-/small-scale farmers; more medium-/small-scale farmers used antibiotics on the sampled birds (OR = 7.70, 95% CI = 2.88–20.61). Furthermore, medium-/small-scale farmers were comparatively less likely to obtain prescriptions (OR = 0.02, 95% CI = 0.00–0.08). This implies that more medium-/small-scale farmers access antibiotics over the counter, promoting abuse and overuse of antimicrobials. This finding indicates a gap in the antimicrobial monitoring of agrovet shops [20]. 

Nevertheless, there was no difference reported between commercial and medium-/small-scale farmers in their being acquainted with the period of withdrawal (OR = 0.96, 95% CI = 0.25–3.74). Generally, 87.3% (103/118) of the farmers had knowledge of the withdrawal period. However, despite this knowledge, 5.8% (6/103) of the knowledgeable farmers admitted to having sold their meat/egg products under treatment. In conformity with studies elsewhere [28,34], our survey showed that more layer farmers sold their products during treatment (OR = 5.38, 95% CI = 1.66–17.42) than broiler farmers. While both broiler and layer farmers have an option to observe the withdrawal period then sell the meat/eggs products, we speculate that layer farmers are tempted to sell their eggs rather than discard them while treating their chickens [35]. This assumption is supported by our data, which showed that most (5/6, 83.3%) of the farmers who knowingly sold products under treatment were layer farmers. Furthermore, more layer farmers than broiler farmers used antibiotics on the sampled birds (OR = 4.23, 95% CI = 1.54–11.63) and for growth promotion (OR = 6.29, 95% CI = 2.00–19.77), probably due to the increasing egg demand [28].

Our study revealed high AMR rates against commonly used antimicrobials such as AMP (128/148, 86.5%) and TET (101/136, 74.3%), similar to findings in other Zambian studies [36,37]. The AMR findings in this study correspond with the Zambian report on AMU by the Ministry of Fisheries and Livestock that cited tetracyclines and penicillins as the most imported antibiotics for administration in animals between 2015 and 2020 [25]. The observed high TET and AMP resistance coincides with the genotypic profile of the representative strains subjected to whole-genome sequencing (WGS), which possessed *tet* and *bla* genes.

While tetracyclines and penicillins are the most used antibiotics in poultry [38], 93.3% (28/30) of the isolates exhibited resistance to three or more drug classes [39]. This could be due to the existence of multiple AMR genes on the same plasmid, which could be co-selected by a single or few drug classes. The observed MDR could be explained by the several identified genes encoding AMR to eight drug classes (Table 2). Importantly, 7.1% (2/28) of MDR strains were resistant to imipenem, a drug of last resort in clinical medicine. However, we found no known carbapenemase-encoding genes, suggesting that the observed resistance could be related to point mutations or novel carbapenemases.

Nonetheless, phenotypic resistance to carbapenems has serious clinical implications as it limits the possible treatment alternatives, especially since colistin is not yet available in Zambian hospitals. The two carbapenem-resistant strains in this study were susceptible to GEN, suggesting that aminoglycosides could be potential treatment options. However, the sample size was too low for a conclusive inference.

In addition to carbapenems, 3GCs have an essential role in clinical practice. This study reported a 20% (30/150) 3GC resistance. Considering the close association between 3GC resistance and ESBLs, we screened our WGS data for various *bla* genes. Previous studies have found the *bla*_CTX-M_ gene in nearly all 3GC-resistant isolates [23,24]. However, only half of the strains in this study harbored the *bla*_CTX-M_ gene (i.e., *bla*_CTX-M-14_, *n* = 2; *bla*_CTX-M-55_, *n* = 2). Meanwhile, the *bla*_TEM_ gene was more prevalent (*n* = 5), while the *bla*_CMY_ existed in three isolates. The presence of *bla*_CMY-2_ genes in 3/8 (37.5%) isolates can be a concern for public health since AmpC *β*-lactamases cause broad-spectrum resistance to *β*-lactamase inhibitors like clavulanic acid [40]. Multi-locus Sequence Type (MLST) identified ST155, among other Sequence Types, similar to (Shawa, et al., 2021) [23], who also found ST155, although their serotypes differed. The presence of AMR genes and plasmids in *E. coli* isolates from poultry may contaminate the environment and food, creating the danger of exposure for humans and animals.

While our study covered five provinces and ten districts, not all samples had epidemiological data. Furthermore, there was no way to verify the questionnaire data as we could not test for antimicrobial residues or physically examine antibiotic sachets or packaging. Also, AMU information could not be verified as we did not have access to prescriptions or antibiotic sales statistics. Having sequenced eight strains, sufficient information was gathered upon which future studies should consider incorporating more samples for a more inclusive picture. Finally, from our short reads data, we could not determine the location of the AMR genes. We therefore implore future studies to consider using a hybrid assembly of short and long reads to provide better accuracy in sequence data quality.

## 4. Materials and Methods

### 4.1. Study Area, Sampling Techniques, Sample Size, and Design

This study was conducted as part of the National Integrated Antimicrobial Resistance Surveillance Strategy (NIAMRSS) [41]. The NIAMRSS is a nationwide human and livestock sector-based cross-sectional study aimed to provide a coherent framework for combating AMR using the “One Health” approach. From this framework, the Protocol on Antimicrobial Resistance Surveillance in Poultry Populations in Zambia 2020–2027 was developed. The nationwide surveillance collects samples from all administrative regions (provinces) through a multistage stratified cluster sampling technique down to districts and farms within the province.

**Figure 2:**
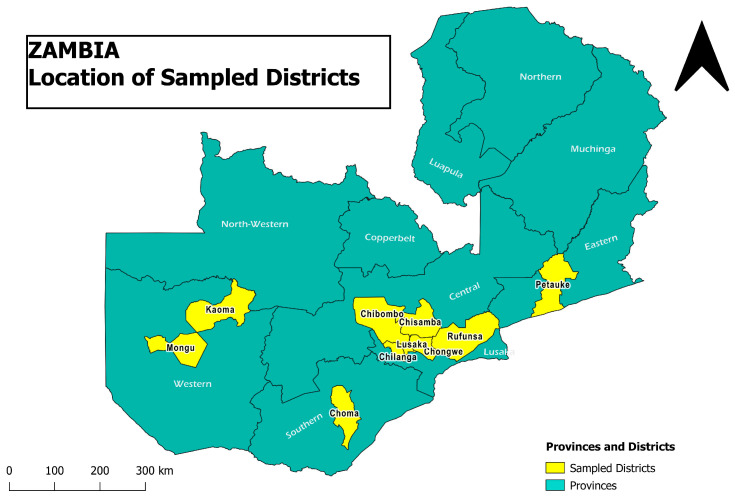
Map of Zambia showing the location of the ten sampled districts.

Based on the strong premise of poultry population, five provinces were purposively selected from which ten districts with at least 5 farms were randomly selected for inclusion in our study districts (Figure 2). Further, the poultry farms within the district were stratified according to the production categories adapted from the Food and Agricultural Organization [42], which divides sectors into classes based on the number of birds per sector. In our study, sector class 1 (≥50,000 birds) and sector class 2 (10,000–49,999 birds) were classified as commercial, while sector class 3 (1000–9999 birds) and sector class 4 (below 1000 birds) were categorized as medium-/small-scale/backyard farming.

The primary sampling unit was a farm, and each poultry house on the farm was considered an independent epidemiological unit. The farms were randomly selected for sample collection and administration of the structured questionnaires between 2019 and 2021. At least five cloacal swabs were collected from each poultry unit and then pooled into a single sample. Samples were collected from poultry units with apparently healthy market-ready birds (four weeks and above for broilers) and at the point of lay for layers.

A total of 269 farms were sampled comprising commercial farms (*n* = 35) and medium-/small-scale farms (*n* = 234). Altogether, the sampled farms included 219 broiler farms and 50 layer farms (Table 4).

Bacterial isolation and phenotypic characterization were performed at the Central Veterinary Research Institute, while molecular analysis was conducted at the University of Zambia, School of Veterinary Medicine. Whole-genome sequencing of ESBL isolates was performed at Noguchi Memorial Institute for Medical Research, University of Ghana.

A total of 119 out of the 269 randomly selected farms consented to participate in the questionnaire survey. A pre-tested structured questionnaire was utilized to collect epidemiological data and information on knowledge, practices, and attitudes on antibiotic use from 35 commercial and 84 medium-/small-scale farmers; 94 of these were broiler farmers and 25 were layer farmers. The questionnaire was pre-tested on 22 poultry farms of Lusaka (*n* = 6), Chilanga (*n* = 8), and Chongwe (*n* = 8) districts by epidemiology and public health specialists from the University of Zambia and the Ministry of Fisheries and Livestock. The study did not incorporate the pilot’s results. The questionnaire had three sections: sample collection and submission, epidemiological, and antimicrobial use sections.

### 4.2. Ethical Approval and Informed Consent

The study was approved by the Excellence in Research Ethics and Science, ERES Converge Ethics Committee (Reference number. 2023-Feb-002). Permission to use the archived isolates was sought from the Ministry of Fisheries and Livestock. Further, the study was cleared by the University of Zambia, School of Veterinary Medicine Board of Graduate Studies Committee. Participants gave their written consent to participate in the study.

### 4.3. Identification and Antimicrobial Susceptibility Testing of E. coli

Well-labelled sterile swab sticks in a Cary-Blair transport medium (Oxoid, Basingstoke, Hampshire, UK) and biohazard bags were used for the aseptic collection of cloacal swabs. The swabs were processed, and *E. coli* was identified as described by Mwaba et al. [22]. The AST was carried out using the Kirby-Bauer disk diffusion method [43] and interpreted using the Clinical and Laboratory Standards Institute (CLSI) guidelines [44].

### 4.4. Phenotypic and Genotypic Detection of ESBL

To determine cefotaxime resistance of the *E. coli* isolates, a total of 150 *E. coli* isolates from December 2019 to August 2021 were inoculated on MacConkey agar (Oxoid, Basingstoke, Hampshire, UK), supplemented with 1 µg/mL of cefotaxime and incubated at 37 °C for 18 h. Next, the broth microdilution method was used to determine the Minimum Inhibitory Concentration (MIC) of these *E. coli* isolates. A single colony was transferred to cefotaxime-supplemented Luria–Bertani broth, incubated for 18 h. The overnight growth cultures were diluted 10^4^-fold and added in triplicates of a serial dilution of cefotaxime in a 96-well plate before incubation at 37 °C for 18 h [45,46].

For detailed characterization of the strains, WGS of ESBL isolates with a MIC ≥ 4 µg/mL was performed using the Illumina NextSeq platform (Illumina Inc., San Diego, CA, USA). Genomic DNA was extracted from 24 h cultured isolates using the QIAamp DNA Mini Kit (QIAGEN Inc. GmbH, Holden, Germany) following the manufacturer’s instructions. The Qubit 4.0 fluorometer assay kit (Thermo Fisher Scientific, Boston, MA, USA) was used to quantify the concentrations of the extracted DNA. Subsequently, the DNA was diluted to achieve concentrations ranging between 10 and 60 ng/µL in a final volume of 30 uL. Libraries of the DNA were prepared using the Illumina DNA library prep–(M) Tagmentation kit (Illumina Inc. San Diego, CA, USA). Using the Agilent 2100 bioanalyzer system (Santa Clara, CA, USA) and the qPCR kappa library quantification kit (Roche, Porterville, CA, USA), respectively, the quality and concentration of fragmented libraries were assessed. The libraries were pooled together and sequenced using a 2 × 150 paired-end method on an Illumina NextSeq platform (Illumina Inc., San Diego, CA, USA). The raw sequencing reads (fastq files) obtained were quality-filtered to a Phred score ≥ 20 and adaptor-trimmed using Trimmomatic (http://www.usadellab.org/cms/index.php?page=trimmomatic, accessed on 17 August 2023 ) [47,48]. The FastQC tool was used to assess the quality of reads (https://www.bioinformatics.babraham.ac.uk/projects/fastqc/, accessed on 17 August 2023). Using the Unicycler assembler v0.5.0, the resulting high-quality reads were de novo assembled into contigs. The quality of the assembled genomes was assessed with Quast v5.2.0. Genomes with coverage exceeding 30X and contigs fewer than 300 bases were selected for post-sequencing analysis. The sequences have been deposited in the Genbank under the BioProject identifier accession number PRJDB17552.

### 4.5. Data Analysis

The collected data were entered into Microsoft Excel MS Office 2019 (Microsoft, Redmond, Washington, DC, USA) for antimicrobial use and WHOnet for antimicrobial susceptibility testing, respectively. These data sets were statistically analyzed using the epiR statistical package in R version 4.2.1. The tools on the Center for Genomic Epidemiology platform were used for post-WGS sequencing analysis to identify resistance genes using Resfinder (https://cge.cbs.dtu.dk/services/ResFinder/, accessed on 23 September 2023), plasmids using Plasmidfinder (https://cge.cbs.dtu.dk/services/PlasmidFinder/, accessed on 23 September 2023), and sequence types using MLSTFinder (https://cge.cbs.dtu.dk/services/MLST/, accessed on 23 September 2023).

## 5. Conclusions

The questionnaire survey results from this study indicate that medium/small-scale farmers used more antibiotics but fewer prescriptions than commercial farmers. The laboratory results showed higher phenotypic ESBL prevalence among medium/small-scale farmers compared to commercial farms. Most *E. coli* isolates obtained from both commercial and medium/small-scale farms exhibited MDR, and WGS revealed *β*-lactamase (*bla*_CMY-2_, *bla*_CTX-M-14_, *bla*_CTX-M-55_, and *bla*_TEM_) and several other AMR genes. These AMR genes pose a health risk as they can potentially be transferred from poultry to the environment and bacterial groups in humans. There is a need to promote the establishment of initiatives that encourage prudent antimicrobial use like farmer field schools in the poultry industry alongside continued AMR/AMU surveillance.

## Figures and Tables

**Table 1:** Comparison of antibiotic use among the commercial and medium-/small-scale farmers from questionnaire survey results.

Variable	Overall Use	Commercial (Reference)	Medium-/Small-Scale	Odds Ratio (OR)	95% CI of OR
Use on sampled birds	57/118 (48.3%)	6/35 (17.1%)	51/83 (61.4%)	7.70	**2.88–20.61**
Prescription use	42/108 (38.9%)	27/29 (93.1%)	15/79 (19.0%)	0.02	**0.00–0.08**
Prophylaxis	23/119 (19.3%)	1/35 (2.9%)	22/84 (26.2%)	12.06	**1.56–93.45**
Growth promotion	15/119 (12.6%)	0/35 (0.0%)	15/84 (17.9%)	7.61	0.97–59.98
Knowledge of the withdrawal period	103/118 (87.3%)	32/35 (91.4%)	71/83 (85.5%)	0.55	0.15–2.10
Sale of products under treatment	14/116 (12.1%)	3/35 (8.6%)	11/81 (13.6%)	1.68	0.44–6.42

Note: Significant results in bold font.

**Table 2:** Detected AMR genes by percentage proportion in *E. coli isolates*.

Antibiotic Class	Detected AMR Genes	Gene Percent Proportion
Aminoglycosides	*aac(3)-Iia*, *aac(3)-Iid*, *aac(3)-Via* *aadA1*, *aadA5* *aph(3′)-Ia*, *aph(3″)-Ib*, *aph(6)-Id*	(3/8, 37.5%) (2/8, 25%) (3/8, 37.5%)
*β*-lactams	*bla*_CTX-M-14_ *bla*_CTX-M-55_ *bla*_TEM_ *bla*_CMY-2_	(2/12, 16.7%) (2/12, 16.7%) (5/12, 41.7%) (3/12, 25%)
Folate-pathway antagonists	*dfrA7*, *dfrA14*, *dfrA17* *sul1*, *sul2*	(3/5, 60%) (2/5, 40%)
Phenicols	*floR*	(1/1, 100%)
Macrolide	*mph(A)*	(1/1, 100%)
Fosfomycin	*fosA3*, *fosA7*	(2/2, 100%)
(Fluoro)quinolones	*qnrB19*, *qnrS1* *OqxA*, *OqxB*	(2/4, 50%) (2/4, 50%)
Tetracycline	*tet(A)* *tet(B)*	(1/2, 50%) (1/2, 50%)

**Table 3:** Distribution and genetic characteristics of sequenced *E. coli* isolates.

S/No	Isolate ID	Location	Farm Type	Sequence Type	OH Serotype	AMR Genes	Plasmids
1	L4F65S1	Petauke	medium-/ small-scale	ST770	O25H16	*aph(3″)-Ib*, *aph(6)-Id*, *bla_CMY-2_*, *floR*, *sul2*, *tet(A)*	IncFIB(AP001918), IncFII, IncB/O/K/Z, p0111
2	L5F6S1	Mongu	medium-/ small-scale	ST117	O45H4	*aadA5*, *aph(3″)-Ib*, *aph(6)-Id*, *bla_CTX-M-55_*, *bla_TEM_*, *dfrA14*, *dfrA17*, *tet(A)*	ColpVC, IncFII(pHN7A8), IncI2
3	L1F154S9	Chisamba	Commercial	ST7938	O32H35	*aac(3)-IIa*, *aadA5*, *aph(3″)-Ib*, *aph(6)-Id*, *bla_CTX-M-14_*, *dfrA17*, *floR*, *fosA3*, *mph(A)*, *OqxB*, *OqxA*, *qnrS1*, *sul1*, *sul2*, *tet(A)*, *tet(B)*	IncFIB(AP001918), IncFIC(FII), IncFII(pHN7A8)
4	L1F8S1	Lusaka	medium-/ small-scale	ST155	O154H51	*aph(3ȃ2)-Ia*, *aph(3″)-Ib*, *aph(6)-Id*, *bla_CTX-M-14_*, *bla_TEM_*, *dfrA7*, *floR*, *fosA3*, *fosA7*, *sul1*, *sul2*, *tet(A)*	IncHI2, IncHI2A, p0111
5	L1F5S1	Lusaka	medium-/ small-scale	ST847	O108H2	*aph(3″)-Ib*, *aph(6)-Id*, *bla_TEM_*, *fosA7*, *qnrS1*, *sul2*, *tet(A)*	IncFII, IncHI2, IncHI2A, IncN, p0111
6	L1F151S5	Chisamba	Commercial	ST211	O22H7	*aac(3)-IId*, *aph(3″)-Ib*, *aph(6)-Id*, *bla_CMY-2_*, *bla_TEM_*, *sul2*, *tet(A)*, *tet(B)*	IncFIB(K), p0111
7	L1F151S3	Chisamba	Commercial	ST770	O102H51	*bla_CMY-2_*	IncI1-I(Alpha), IncX4
8	L1F152S5	Chisamba	Commercial	ST770	O102H51	*aac(3)-Via*, *aadA1*, *bla*_CTX-M-55_, *bla*_TEM_, *fosA3*, *qnrB19*, *sul1*, *sul2*	IncFIB(AP001918), IncFII, IncN

**Table 4:** Sample distribution by district, production scale, and type.

S/No	District	Samples	Commercial	Medium/Small	Broiler	Layer
1	Chibombo	10	0	10	9	1
2	Chilanga	44	0	44	30	14
3	Chisamba	81	32	49	75	6
4	Choma	13	0	13	9	4
5	Chongwe	43	0	43	37	6
6	Kaoma	12	0	12	12	0
7	Lusaka	30	3	27	16	14
8	Mongu	13	0	13	12	1
9	Petauke	18	0	18	14	4
10	Rufunsa	5	0	5	5	0
	Total	269	35	234	219	50

## Data Availability

The supporting data of this manuscript can be made available on request from the corresponding author.

## Supplementary Materials

The following supporting information can be downloaded at: https://www.mdpi.com/article/10.3390/antibiotics13050467/s1. Table S1. Questionnaire survey results on use of antibiotics among Layer and broiler the farmers in the study population; Table S2 Antibiotic Susceptibility Pattern of suspected ESBL-Producing E. coli on cefotaxime screening by district.
