# Supplementary material for: Antimicrobial Use Survey and Detection of ESBL-Escherichia coli in Commercial and Medium-/Small-Scale Poultry Farms in Selected Districts of Zambia
Source: Antibiotics (Basel). 2024 May 20;13(5):467. doi: 10.3390/antibiotics13050467 (PMC11118926; doi:10.3390/antibiotics13050467)
Supplement: Supplementary file 1 [file antibiotics-13-00467-s001.zip › antibiotics-2889481-supplementary.pdf]

Supplementary Materials

Table S1. Questionnaire survey results on use of antibiotics among Layer and broiler the farmers in the study population.

Variable	Overall Use	Broiler (Reference)	Layer	Odds ratio (OR)	95% CI
Use on sampled birds	57/118 (48.3%)	39/94 (41.5%)	18/24 (75.0%)	4.23	**1.54–11.63**
Prescription use	42/108 (38.9%)	33/87 (37.9%)	9/21 (42.9%)	1.23	0.47–3.23
Prophylaxis	23/119 (19.3%)	15/95 (15.8%)	8/24 (33.3%)	2.67	0.97–7.34
Growth promotion	15/119 (12.6%)	7/95 (7.4%)	8/24 (33.3%)	6.29	**2.00–19.77**
Knowledge of withdrawal period	103/118 (87.3%)	83/95 (87.4%)	20/23 (87.0%)	0.96	0.25–3.74
Sale of products under treatment	14/116 (12.1%)	7/93 (7.5%)	7/23 (30.4%)	5.38	**1.66–17.42**

Note: Significant results in bold font.

Table S2. Antibiotic Susceptibility Pattern of suspected ESBL-Producing *E. coli* on cefotaxime screening by district.

District	Number of Isolates	Phenotypic ESBL positives isolates	CTX MIC values (µg/mL)					
			**2**	**4**	**8**	**16**	**32**	**≥64**
Chisamba	51	10 (19.6%)	6	0	1	0	1	2
Mongu	15	2 (13.0%)	1	0	0	0	0	1
Lusaka	32	4 (12.5%)	2	1	1	0	0	0
Choma	13	2 (15.3%)	2	0	0	0	0	0
Petauke	10	3 (30.0%)	1	1	0	0	0	1
Chibombo	3	0 (0.0%)	0	0	0	0	0	0
Chilanga	3	2 (66.7%)	2	0	0	0	0	0
Chongwe	12	6 (50.0%)	6	0	0	0	0	0
Rufunsa	3	0 (0.0%)	0	0	0	0	0	0
Kaoma	8	1 (12.5%)	1	0	0	0	0	0
Total	150	30 (20.0%)	21	2	2	0	1	4

CXT MIC-Cefotaxime Minimum Inhibitory Concentration
